# Supplementary material for: High-resolution visualization of H3 variants during replication reveals their controlled recycling
Source: Nat Commun. 2018 Aug 9;9:3181. doi: 10.1038/s41467-018-05697-1 (PMC6085313; doi:10.1038/s41467-018-05697-1)
Supplement: Supplementary file 1 — Supplementary Information [file 41467_2018_5697_MOESM1_ESM.pdf]

Supplementary Information

High-resolution visualization of H3 variants during replication reveals their controlled recycling

Clément et al.

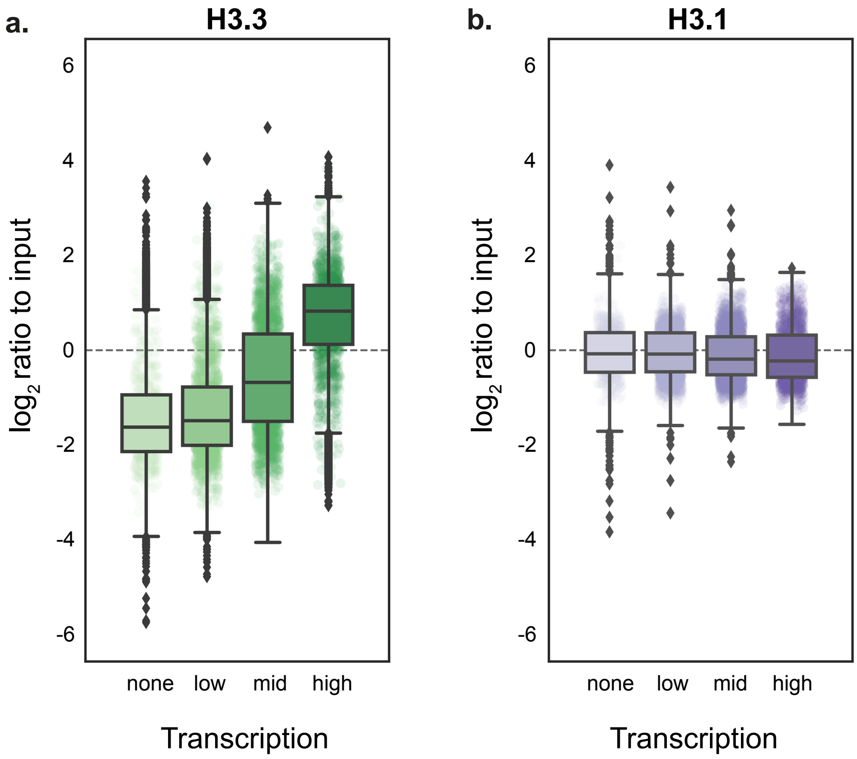

Supplementary Figure 1

a. H3.3 distribution by transcription levels. For a and b, the box plots show, respectively, the distribution of H3.3 and H3.1 log_2 ratios to input (y-axis) at 10 kb regions ranked by transcriptional status (x-axis). The rank reflects the level of nascent transcription, from none to low (lower 10th percentile), mid (10th to 90th percentile) or high (upper 10th percentile) (highlighted with increasingly darker colors).
b. H3.1 distribution by transcription levels.

Supplementary Figure 2

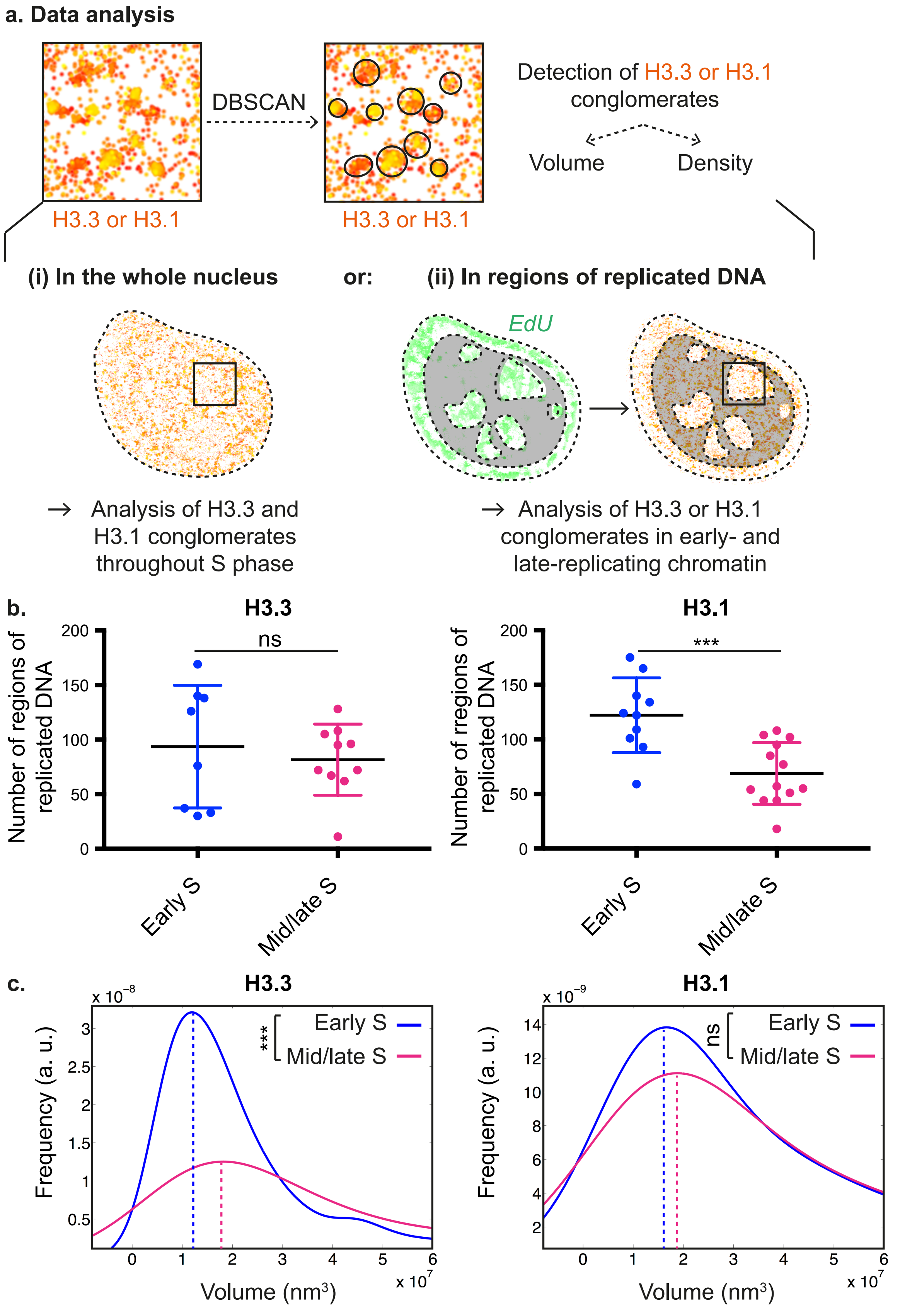

a. Analysis of the H3.3 or H3.1 signal. The DBSCAN clustering algorithm groups together detections with enough neighbors in a given radius. We refer to the isolated clusters as H3.3 or H3.1 conglomerates. We analyze the volume and density of these conglomerates.
b. Number of regions or replicated DNA detected using the DBSCAN approach applied to the EdU signal in our HeLa H3.3- (left) or H3.1-SNAP (right) cell lines. Error bars represent standard deviation. p-values (using a t test): (***) p≤0.001; (**) p≤ 0.01; (*) p≤ 0.05; (ns) not significant.
c. The plots show the distribution of volumes of regions of replicated DNA in our HeLa H3.3- (left) or H3.1-SNAP (right) cell lines. Error bars represent standard deviation. p-values (using a Mann-Whitney-Wilcoxon test): (***) p≤0.001; (**) p≤ 0.01; (*) p≤ 0.05; (ns) not significant.

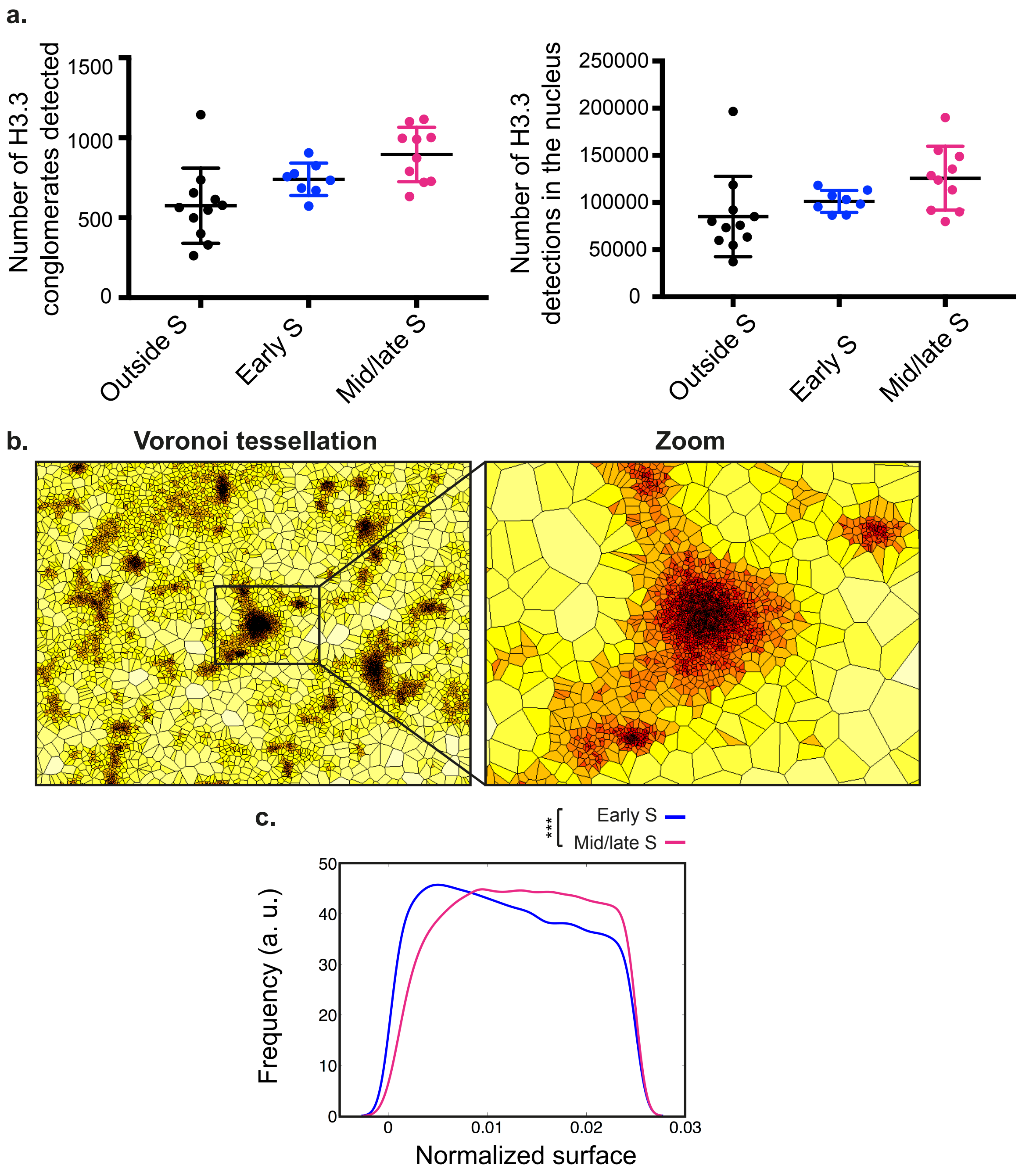

Supplementary Figure 3

a. (Left) Number of H3.3 conglomerates detected; (right) total number of H3.3 detections in the nucleus. 20 000 frames were used. Error bars represent standard deviation.
b. Voronoi tessellation of STORM data. This partitions the signal into polygons such that each polygon contains a single detection. Large polygons (yellow) correspond to low-density regions and small polygons (dark red) correspond to high-density regions.
c. Voronoi surface analysis: the plot shows the distribution of small surfaces in cells in early S phase (blue) and in mid/late S phase (magenta). For early S, N=8 cells and, for mid/late S, N=10 cells. p-values (using a Mann-Whitney-Wilcoxon test): (***) p≤0.001; (**) p≤ 0.01; (*) p≤ 0.05; (ns) not significant.

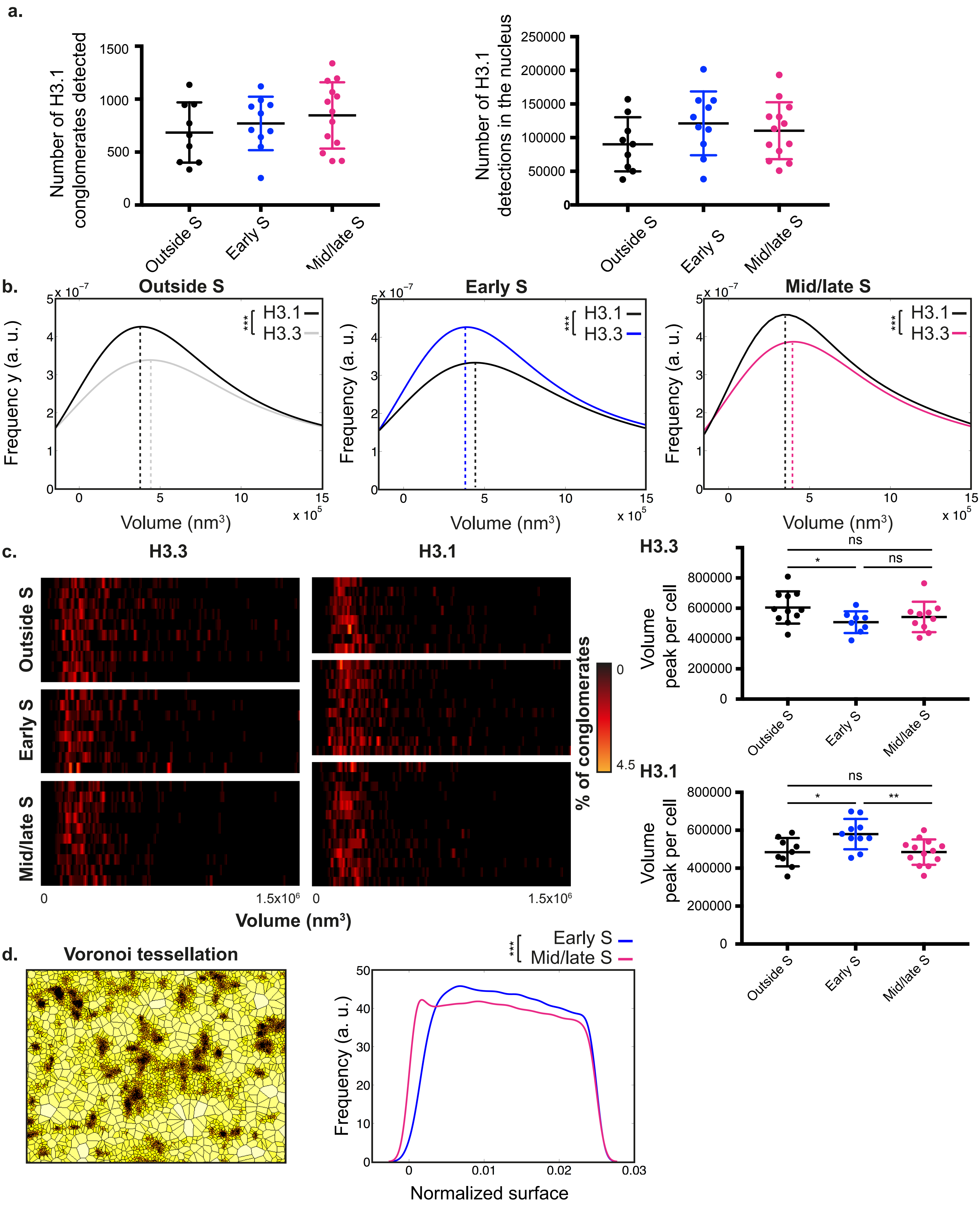

a.
Number of H3.1 conglomerates detected
Number of H3.1 detections in the nucleus
Outside S
Early S
Mid/late S
b.
Outside S
Early S
Mid/late S
x 10−7
Frequenc y (a. u.)
Frequency (a. u.)
H3.1
H3.3
***
Volume (nm3)
x 105
c.
H3.3
H3.1
Outside S
Early S
Mid/late S
% of conglomerates
0
4.5
0
1.5x106
Volume (nm3)
Volume peak per cell
ns
*
**
d.
Voronoi tessellation
Early S
Mid/late S
Frequency (a. u.)
Normalized surface

Supplementary Figure 4

a. (Left) Number of H3.1 conglomerates detected; (right) total number of H3.1 detections in the nucleus. 20 000 frames were used. Error bars represent standard deviation.

b. The plots show the distributions of the volume of H3.3 or H3.1 conglomerates in cells outside S phase (left), in early S phase (middle), and in mid/late S phase (right).

c. Conglomerate volume distributions in individual cells. (Left) Each row represents a single cell where the percentage of conglomerates falling in every 7500 nm^3 bin was calculated and normalized for display. Heatmaps are unsorted within each group. (Right) Scatter plot of the corresponding volume peaks cell by cell. Error bars represent standard deviation. p-values (using a t test): (***) $p \leq 0.001$; (**) $p \leq 0.01$; (*) $p \leq 0.05$; (ns) not significant. Error bars represent standard deviation.

d. (Left) Voronoi tessellation of STORM data. This partitions the signal into polygons such that each polygon contains a single detection. Large polygons (yellow) correspond to low-density regions and small polygons (dark red) correspond to high-density regions. (Right) Voronoi surface analysis: the plot shows the distribution of small surfaces in cells in early S phase (blue) and in mid/late S phase (magenta). For early S, N=10 cells and for mid/late S, 13 cells. p-values (using a Mann-Whitney-Wilcoxon test): (***) $p \leq 0.001$; (**) $p \leq 0.01$; (*) $p \leq 0.05$; (ns) not significant. Error bars represent standard deviation.

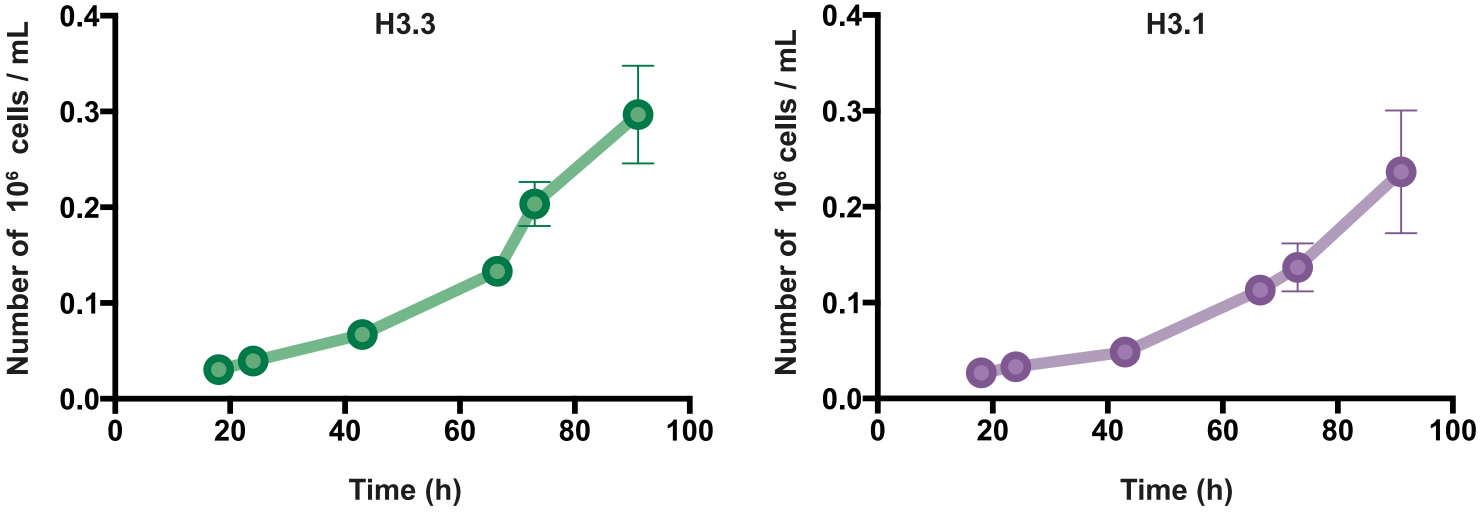

Supplementary Figure 5

Growth curves corresponding to the two cell lines HeLa H3.3- and H3.1-SNAP. Error bars represent standard deviation. Three experimental replicates were used.

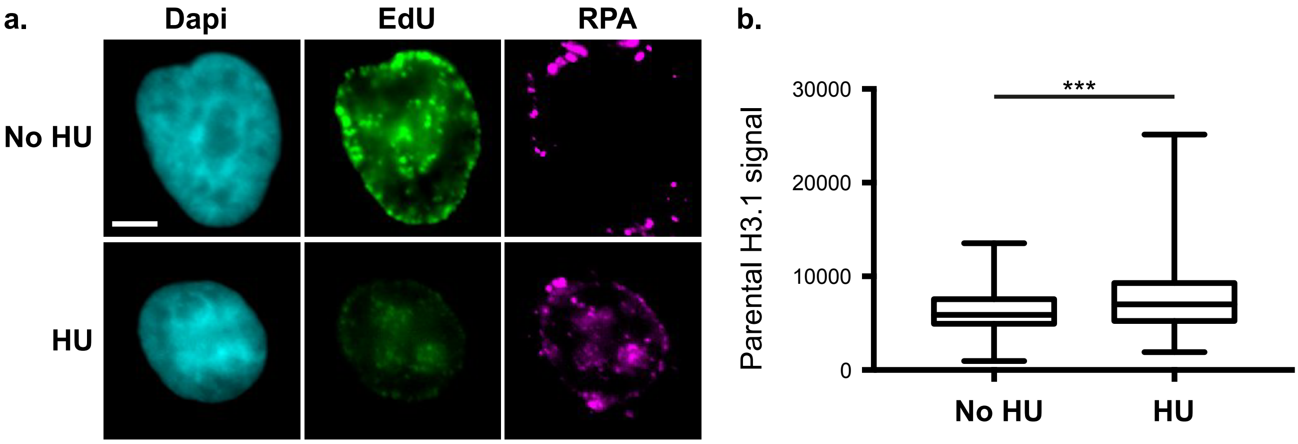

Supplementary Figure 6

a. RPA staining (magenta) and EdU staining (green) in control and HU-treated conditions, revealed by immunofluorescence. DAPI stains nuclei (cyan). The images were acquired using an epifluorescence microscope. Scale bars represent 5 μm.
b. Quantification of parental H3.1 signal using epifluorescence microscopy in the control and HU-treated condition. p-values (using a t test): (***) p≤0.001; (**) p≤ 0.01; (*) p≤ 0.05; (ns) not significant.

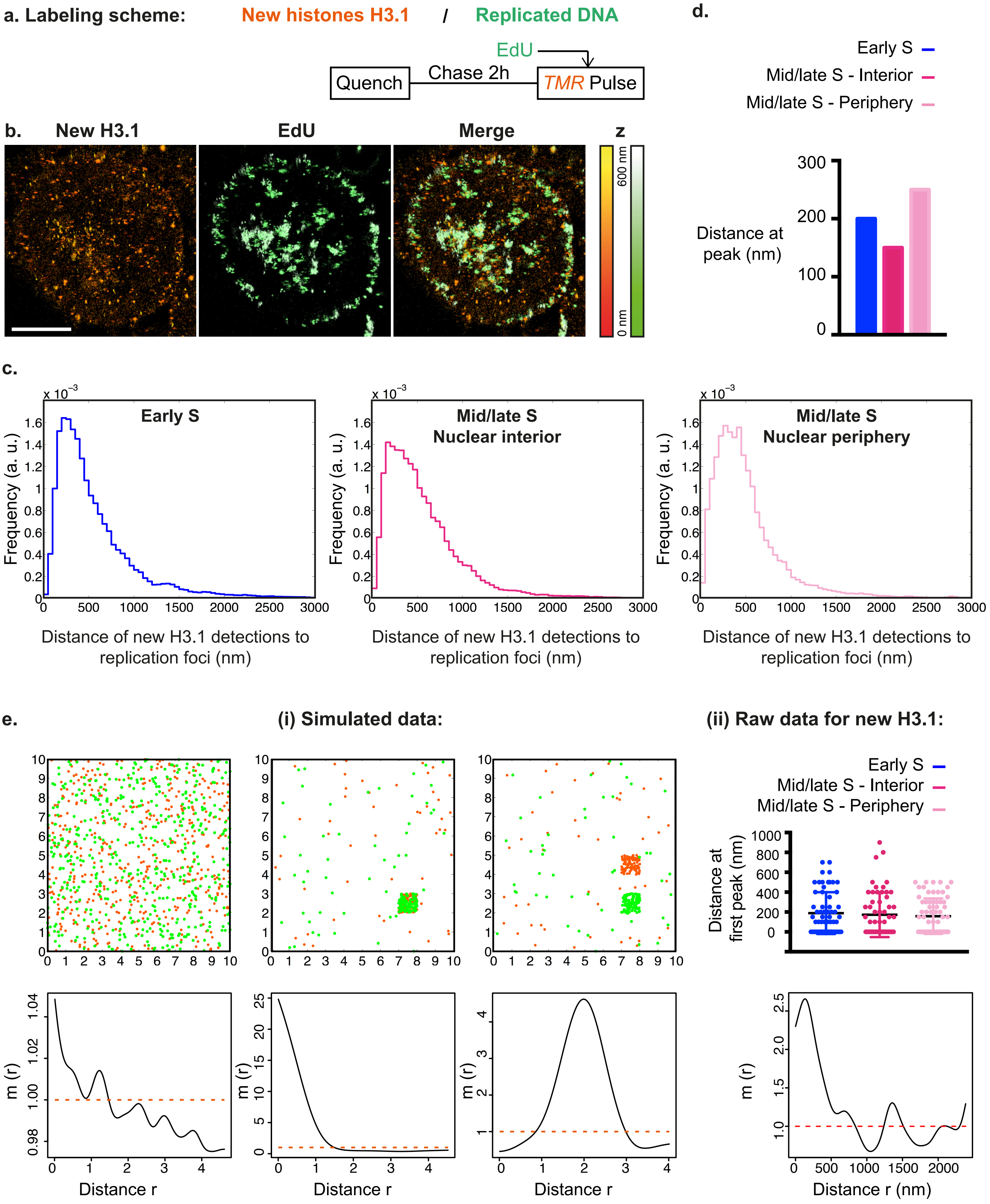
a. Labeling scheme:
New histones H3.1
/
Replicated DNA
EdU
Quench
Chase 2h
TMR Pulse
b. New H3.1
EdU
Merge
z
600 nm
0 nm
c.
Early S
Mid/late S
Nuclear interior
Mid/late S
Nuclear periphery
Frequency (a. u.)
Distance of new H3.1 detections to replication foci (nm)
d.
Early S
Mid/late S - Interior
Mid/late S - Periphery
Distance at peak (nm)
e.
(i) Simulated data:
(ii) Raw data for new H3.1:
Early S
Mid/late S - Interior
Mid/late S - Periphery
Distance at first peak (nm)
m (r)
Distance r
Distance r (nm)

Supplementary Figure 7

a. Labeling scheme using H3.1-SNAP to follow new histones. A quench step labels all preexisting histones with a non-fluorescent dye. A chase step allows synthesis and deposition of new unlabeled H3.1-SNAP. A pulse using the fluorophore TMR (orange) labels H3.1-SNAP. The EdU labeling and triton extraction are as described in Figure 2.

b. Representative STORM images of new H3.1 (TMR, orange) and replicated DNA (EdU, green) in HeLa H3.1-SNAP. The color gradient corresponds to the z range. Scale bars represent 5 µm.

c. The plots show the distributions of the distances of new H3.1 to the center of replicated DNA in early S phase cells (left), mid/late S phase cells in the nuclear interior (middle), and mid/late S phase cells at the nuclear periphery (right).

d. Distance at the peak in the distribution plots in c for cells in early S phase (blue), mid/late S phase cells in the nuclear interior (dark pink), and at the nuclear periphery (light pink).

(i) (Top) Simulated data of two signals for (left) randomly distributed populations, (middle) two colocalizing signals, and (right) two signals at a fixed distance from each other. (Bottom) m function plotting the enrichment of the orange signal relating to the green in the three cases. Region of the graph above 1 (dashed orange line) indicates attraction. (ii) m function applied to the signal of new H3.1 and the signal of EdU. In each cell, we applied the function locally to 8 zones at the nuclear periphery and 8 zones in the nuclear interior. (Bottom) Example of m function for one zone. (Top) The scatter plot indicates the distance at the maximum of the first peak for each zone. Due to low signal in some zones, some m functions did not feature a peak of enrichment and were excluded. Error bars represent standard deviation.

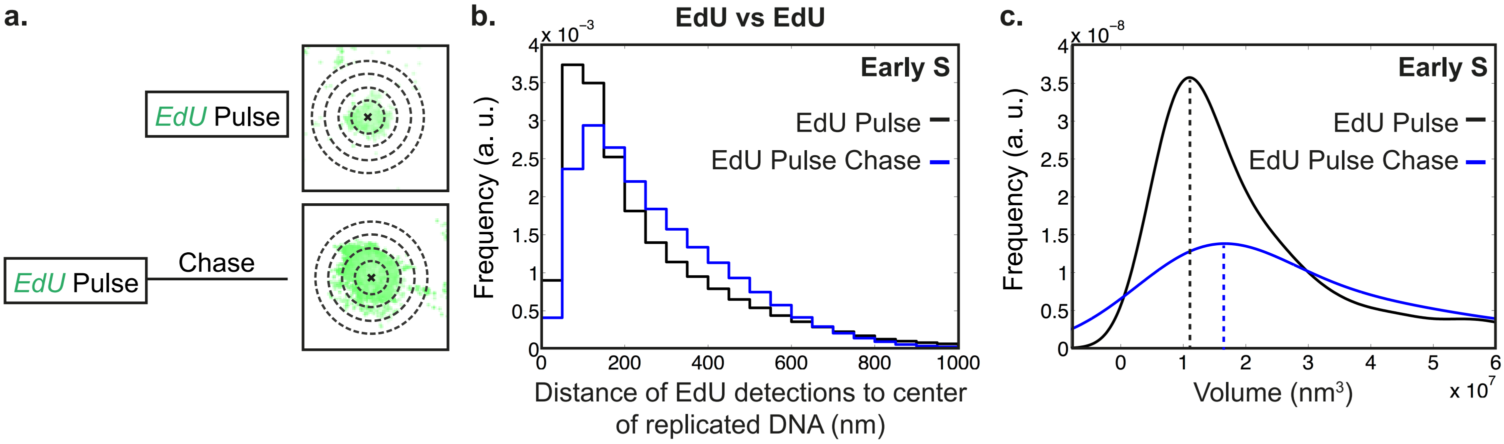

Supplementary Figure 8

a. Scheme of the experiment: we either performed a single EdU pulse (EdU pulse), or an EdU pulse followed by a 30 min-chase (EdU pulse-chase) in HeLa H3.1-SNAP.

b. Distribution plots of the distances of EdU detections to the center of replicated DNA for the EdU pulse (black) and EdU pulse-chase (blue) in early S phase cells.

c. The plot shows the distribution of the volume of regions of replicated DNA in cells for the EdU pulse (black) and EdU pulse-chase (blue) in early S phase cells.

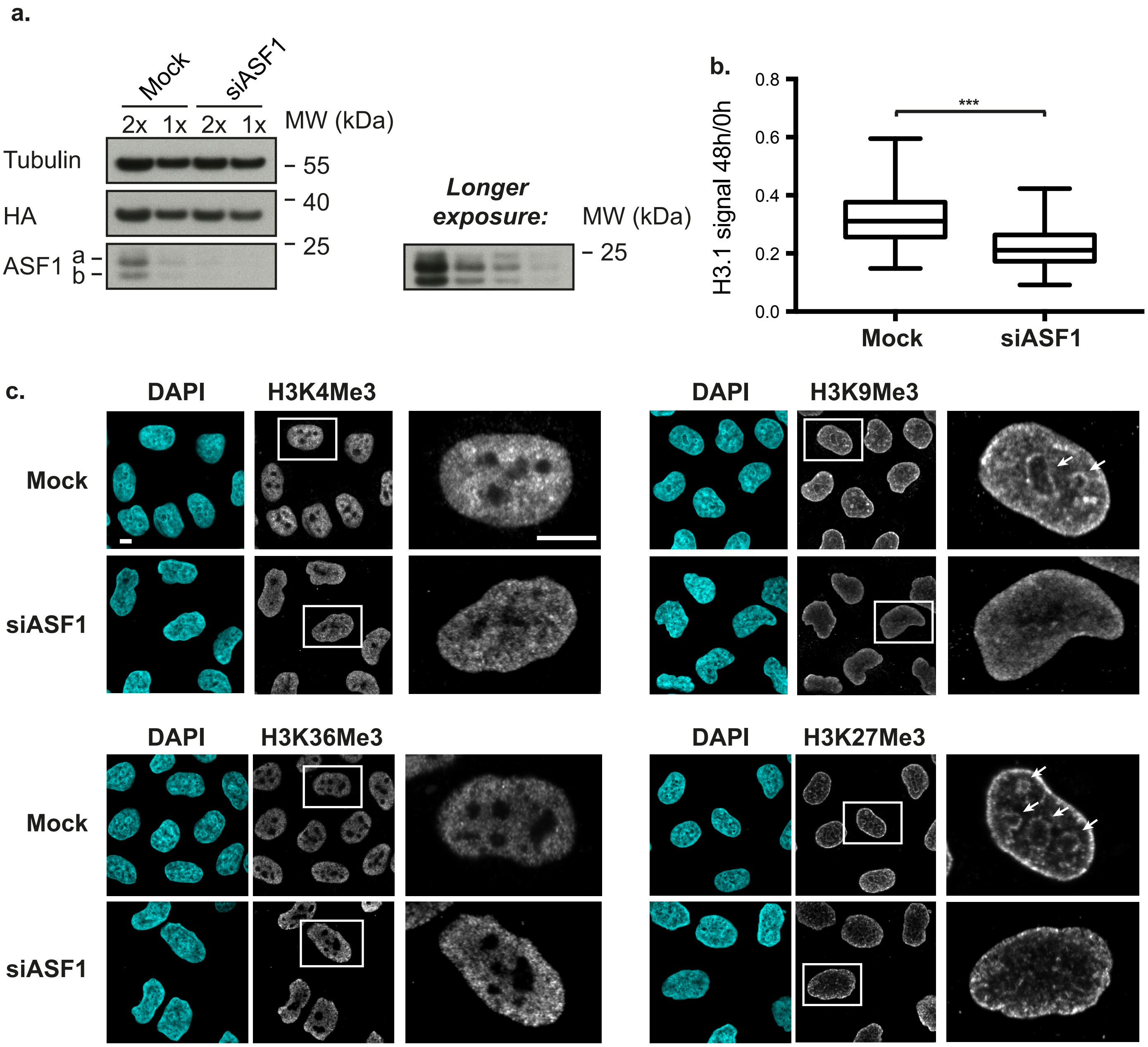

Supplementary Figure 99

a. Western Blot analysis of ASF1a and ASF1b knockdown efficiency using siRNA against ASF1. The SNAP H3.1 also contains a HA-tag, which was probed for in this Western Blot.
b. Ratio between the final (48h-chase after pulse, about two divisions) and the initial (0h after pulse) H3.1 signal in the whole nucleus in mock versus siASF1-transfected cells following a pulse-chase experiment in a synchronized population. An epifluorescence microscope was used. p-values (using a t test): (***) $p \leq 0.001$; (**) $p \leq 0.01$; (*) $p \leq 0.05$; (ns) not significant.
c. H3K4me3, H3K36me3, H3K27me3 and H3K9me3 staining (white) in control and ASF1-depleted conditions, revealed by immunofluorescence in HeLa H3.1-SNAP cells. DAPI stains nuclei (blue). The images were acquired using a confocal microscope. Scale bars represent 10 µm.

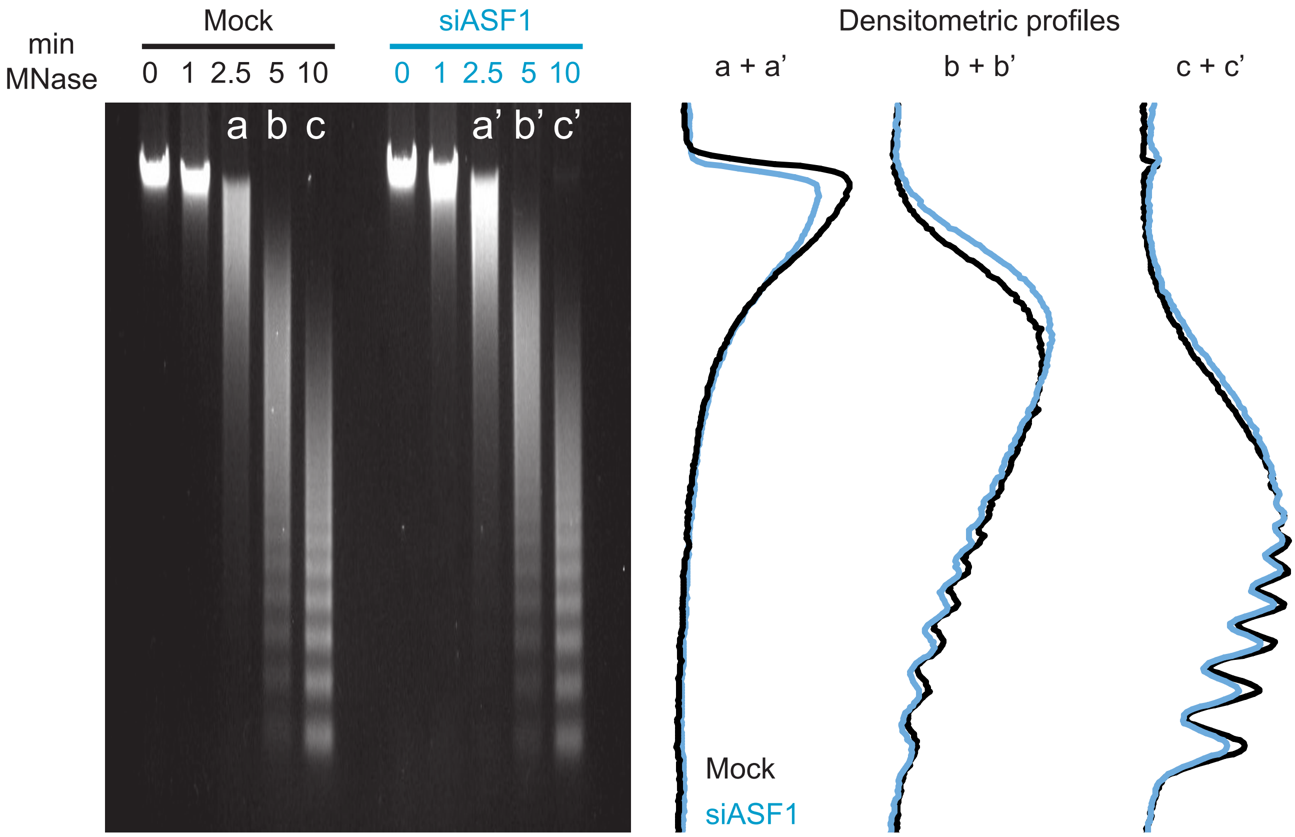

Supplementary Figure 10

MNase sensitivity analysis. Agarose gel electrophoresis of total DNA extracted from Mock or siASF1 cells after the indicated times of MNase treatment (left). Densitometric plots comparing digestion profiles at 2.5, 5 and 10 minutes of digestion show no change between the two conditions (right).

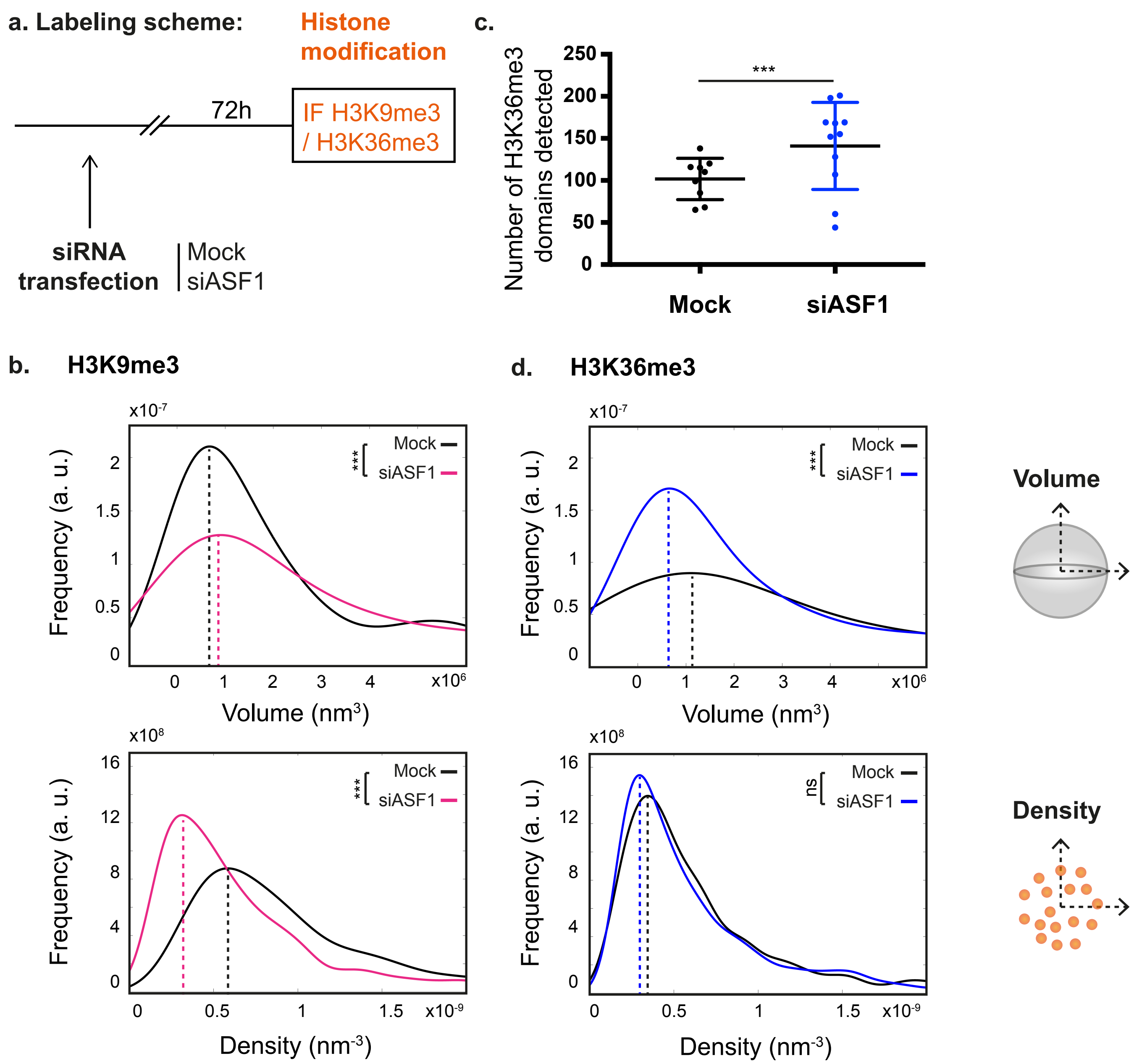

Supplementary Figure 11

a. Labeling scheme for H3K9me3 and H3K36me3 stainings in HeLa H3.1-SNAP in upon siRNA treatment (mock or siASF1).

b. The plots show the distribution of volume (top) or density (bottom) of H3K9me3 domains in cells in early S phase in mock (N=4 cells) and ASF1-depleted cells (N=7 cells). For b and d, p-values (using a Mann-Whitney-Wilcoxon test): (***) $p \leq 0.001$; (**) $p \leq 0.01$; (*) $p \leq 0.05$; (ns) not significant.

c. Number of H3K36me3 domains detected. p-values (using a t test): (***) $p \leq 0.001$; (**) $p \leq 0.01$; (*) $p \leq 0.05$; (ns) not significant. Error bars represent standard deviation.

d. The plots show the distribution of volume (top) or density (bottom) of H3K36me3 domains in cells in early S phase in mock (N=9 cells) and ASF1-depleted cells (N=11 cells).

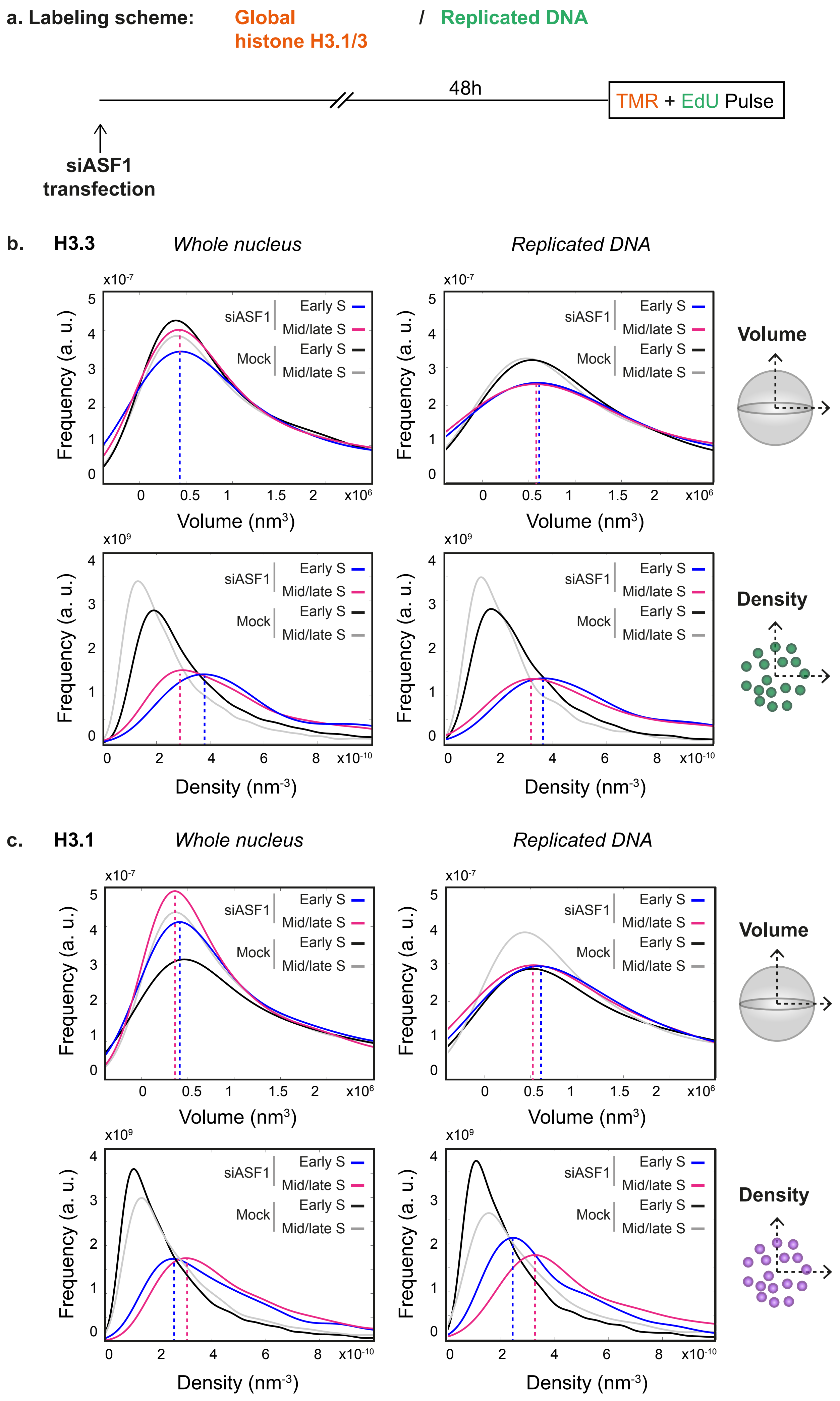
a. Labeling scheme:
Global histone H3.1/3
/
Replicated DNA
48h
TMR + EdU Pulse
siASF1 transfection
b.
H3.3
Whole nucleus
Replicated DNA
x10-7
Frequency (a. u.)
Volume (nm3)
x106
x109
Density (nm-3)
x10-10
siASF1
Mock
Early S
Mid/late S
Volume
Density
c.
H3.1
Whole nucleus
Replicated DNA

Supplementary Figure 12

Global distribution of H3.3/1 upon ASF1 depletion throughout S phase using STORM assay

a. Labeling scheme using H3.3/1-SNAP to follow global H3.3/1 as described in Figure 2 upon siASF1 treatment.

b. (Left) For H3.3- conglomerates in the whole nucleus: the plots show the distribution of volume (top) or density (bottom) of H3.3 conglomerates in cells in early S phase (blue), and in mid/late S phase (magenta) upon ASF1 depletion or in the mock condition (black and grey for early and mid/late, respectively) as in Figure 3. (Right) For H3.3 conglomerates in regions of replicated DNA: the plots show the distribution of volume (top) or density (bottom) of H3.3 conglomerates in cells in early S phase (blue), and in mid/late S phase (magenta) upon ASF1 depletion or in the mock condition (black and grey for early and mid/late, respectively) as in Figure 3. N=6 and 7 cells for early S phase and mid/late S phase respectively upon ASF1 depletion.

c. (Left) For H3.1- conglomerates in the whole nucleus: the plots show the distribution of volume (top) or density (bottom) of H3.1 conglomerates in cells in early S phase (blue), and in mid/late S phase (magenta) upon ASF1 depletion or in the mock condition (black and grey for early and mid/late, respectively) as in Figure 4. (Right) For H3.1 conglomerates in regions of replicated DNA: the plots show the distribution of volume (top) or density (bottom) of H3.1 conglomerates in cells in early S phase (blue), and in mid/late S phase (magenta) upon ASF1 depletion or in the mock condition (black and grey for early and mid/late, respectively) as in Figure 4. N=7 and 9 cells for early S phase and mid/late S phase respectively upon ASF1 depletion.

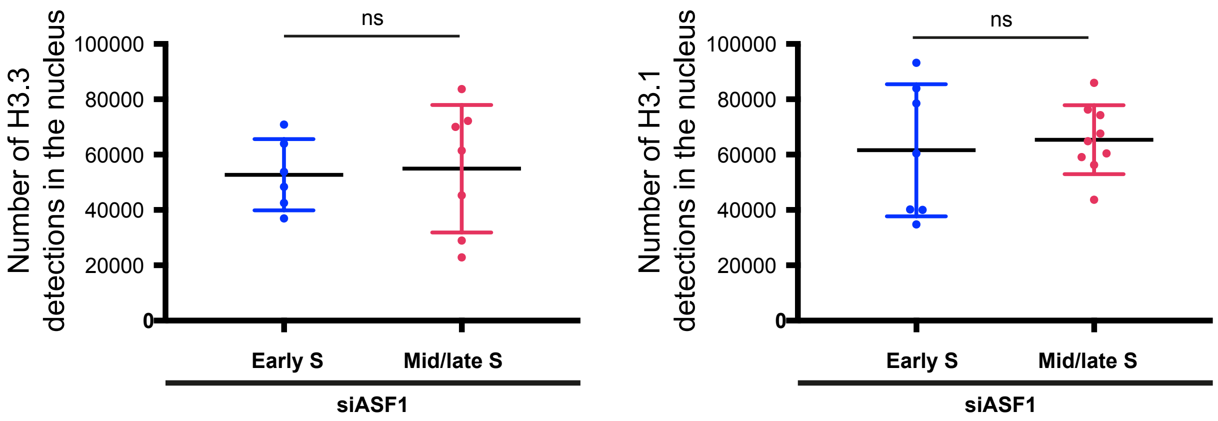

Supplementary Figure 13

Total number of H3.3 (left) or H3.1 (right) detections in the nucleus corresponding to the experiment described in Supplementary Figure 12. 5000 frames were used. p-values (using a t test): (***) p≤0.001; (**) p≤ 0.01; (*) p ≤ 0.05; (ns) not significant. Error bars represent standard deviation.

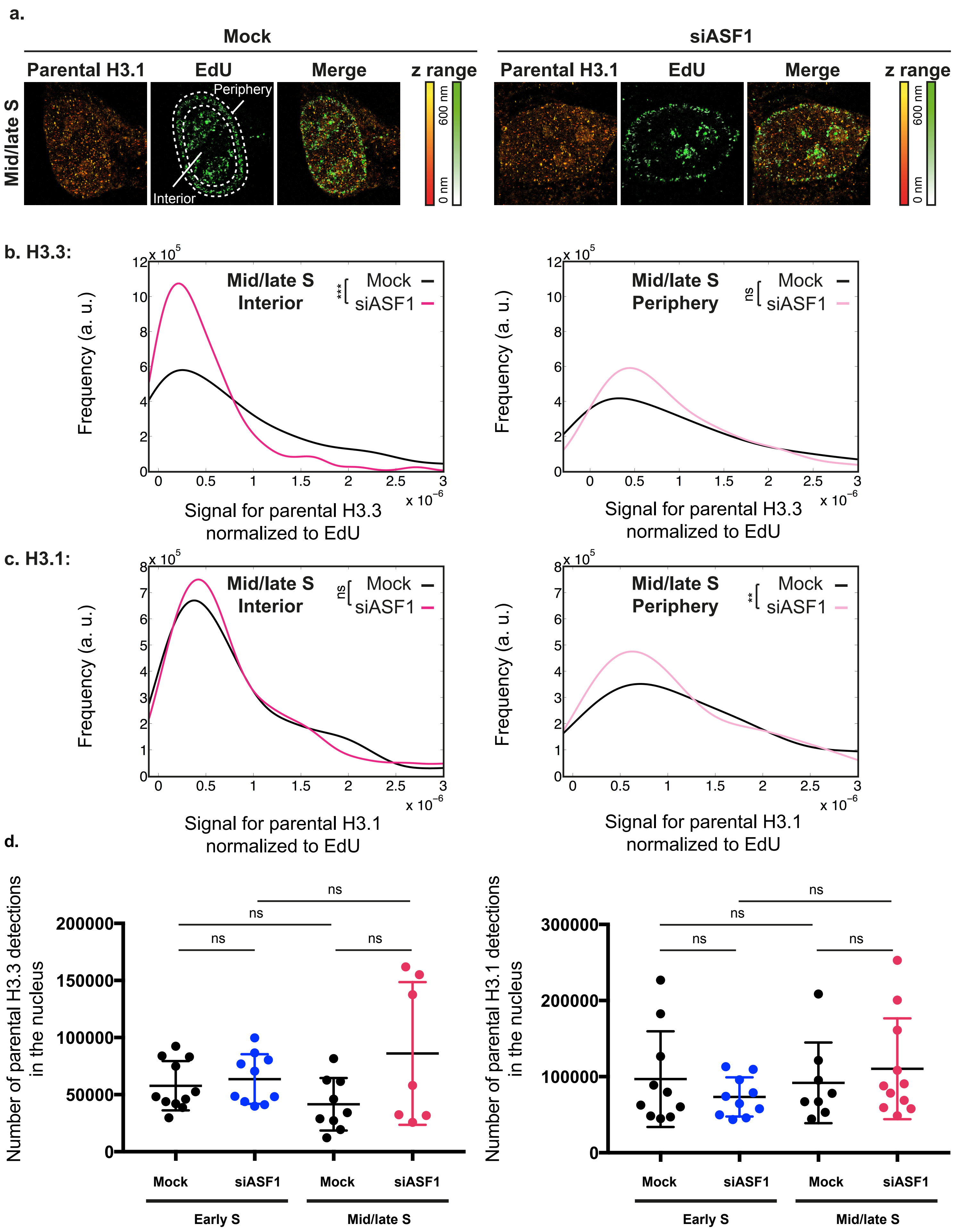
a.
Mock
siASF1
Parental H3.1
EdU
Merge
z range
Mid/late S
Periphery
Interior
600 nm
0 nm
b. H3.3:
Mid/late S
Interior
Mid/late S
Periphery
Mock
siASF1
***
ns
Frequency (a. u.)
x 10^5
x 10^-6
Signal for parental H3.3 normalized to EdU
c. H3.1:
**
Signal for parental H3.1 normalized to EdU
d.
Number of parental H3.3 detections in the nucleus
Number of parental H3.1 detections in the nucleus
Early S
Mid/late S

Supplementary Figure 14

a. Representative STORM images of parental H3.1 (TMR, orange) and replicated DNA (EdU, green) in HeLa H3.1-SNAP in mid/late S phase. The color gradient corresponds to the z range. Scale bars represent 5 µm.

b. The plots show the distributions of the signal for parental H3.3 normalized to EdU in mid/late S phase (left) in the nuclear interior in control (mock, black) and ASF1-depleted cells (siASF1, dark pink). And (right) at the nuclear periphery in control (mock, black) and ASF1-depleted cells (siASF1, light pink). In the mock condition, N=9 cells, and in the siASF1 condition, N=7 cells. For b and c, p-values (using a Mann-Whitney-Wilcoxon test): (***) $p \leq 0.001$; (**) $p \leq 0.01$; (*) $p \leq 0.05$; (ns) not significant.

c. The plots show the distributions of the signal for parental H3.1 normalized to EdU in mid/late S phase (left) in the nuclear interior in control (mock, black) and ASF1-depleted cells (siASF1, dark pink) and (right) at the nuclear periphery in control (mock, black) and ASF1-depleted cells (siASF1). In the mock condition, N=8 cells, and in the siASF1 condition, N=11 cells.

d. Total number of parental H3.3 (left) or H3.1 (right) detections in the nucleus. p-values (using a t test): (***) $p \leq 0.001$; (**) $p \leq 0.01$; (*) $p \leq 0.05$; (ns) not significant. Error bars represent standard deviation.

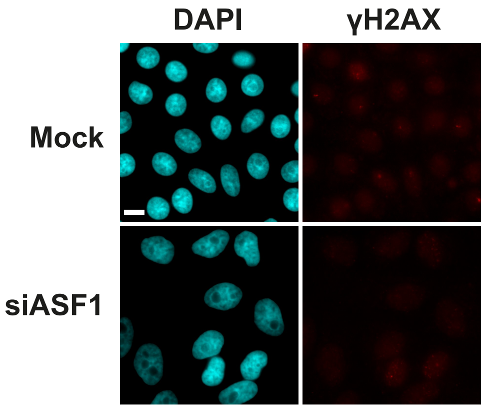

Supplementary Figure 15

γH2AX staining (red) in control and ASF1-depleted conditions, revealed by immunofluorescence in HeLa H3.1-SNAP cells. DAPI stains nuclei (blue). The images were acquired using an epifluorescence microscope. Scale bars represent 10 μm.

a. H3.3

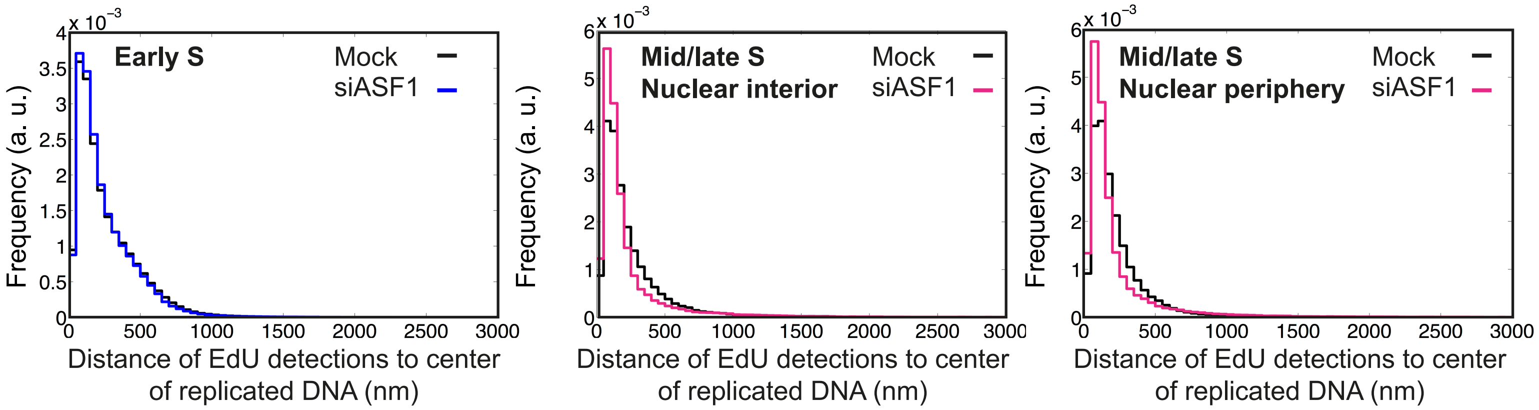

b. H3.1

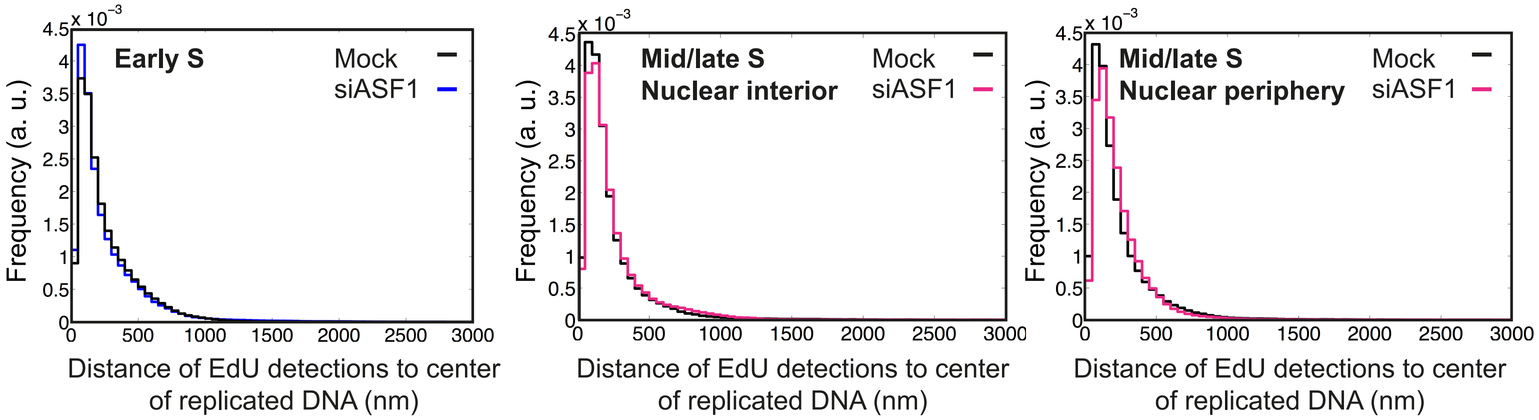

Supplementary Figure 16

a. Spatial distribution of EdU relative to replicated DNA corresponding to experiment described in Figure 8c in HeLa H3.3-SNAP. The plot shows the distribution of the distances of EdU detections to the center of replicated DNA sites for control (black) and ASF1-depleted (blue, pink) cells. The same cells were used as in Figure 8c.

b. Spatial distribution of EdU relative to replicated DNA corresponding to experiment described in Figure 8d in HeLa H3.1-SNAP. The plot shows the distribution of the distances of EdU detections to the center of replicated DNA sites for control (black) and ASF1-depleted (blue, pink) cells. The same cells were used as in Figure 8d.

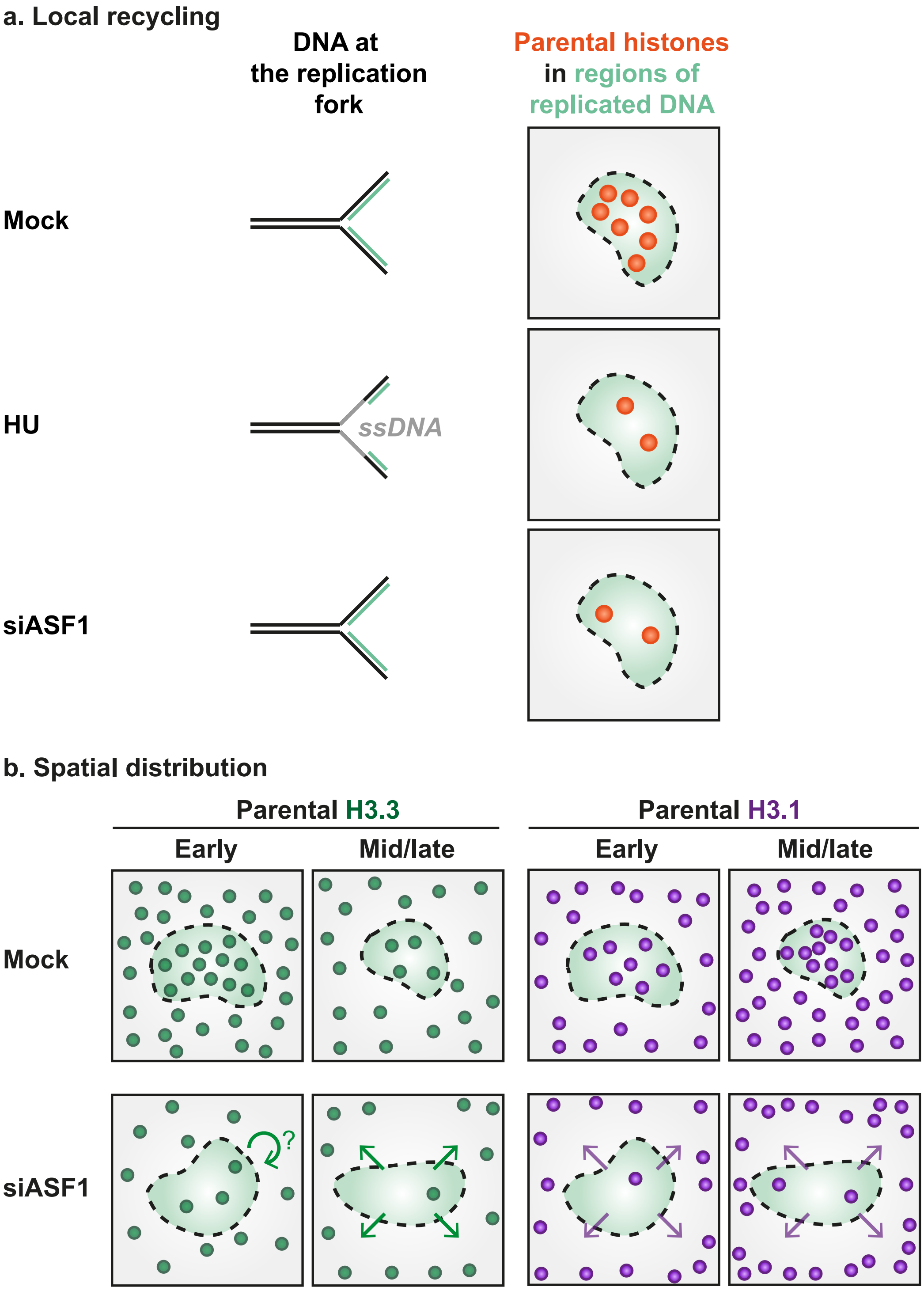

Supplementary Figure 17

a. Scheme summarizing the effect of HU treatment or ASF1 depletion on local recycling of parental histones.
b. Scheme summarizing the effect of ASF1 depletion on spatial distribution of parental histones relative to replication sites.

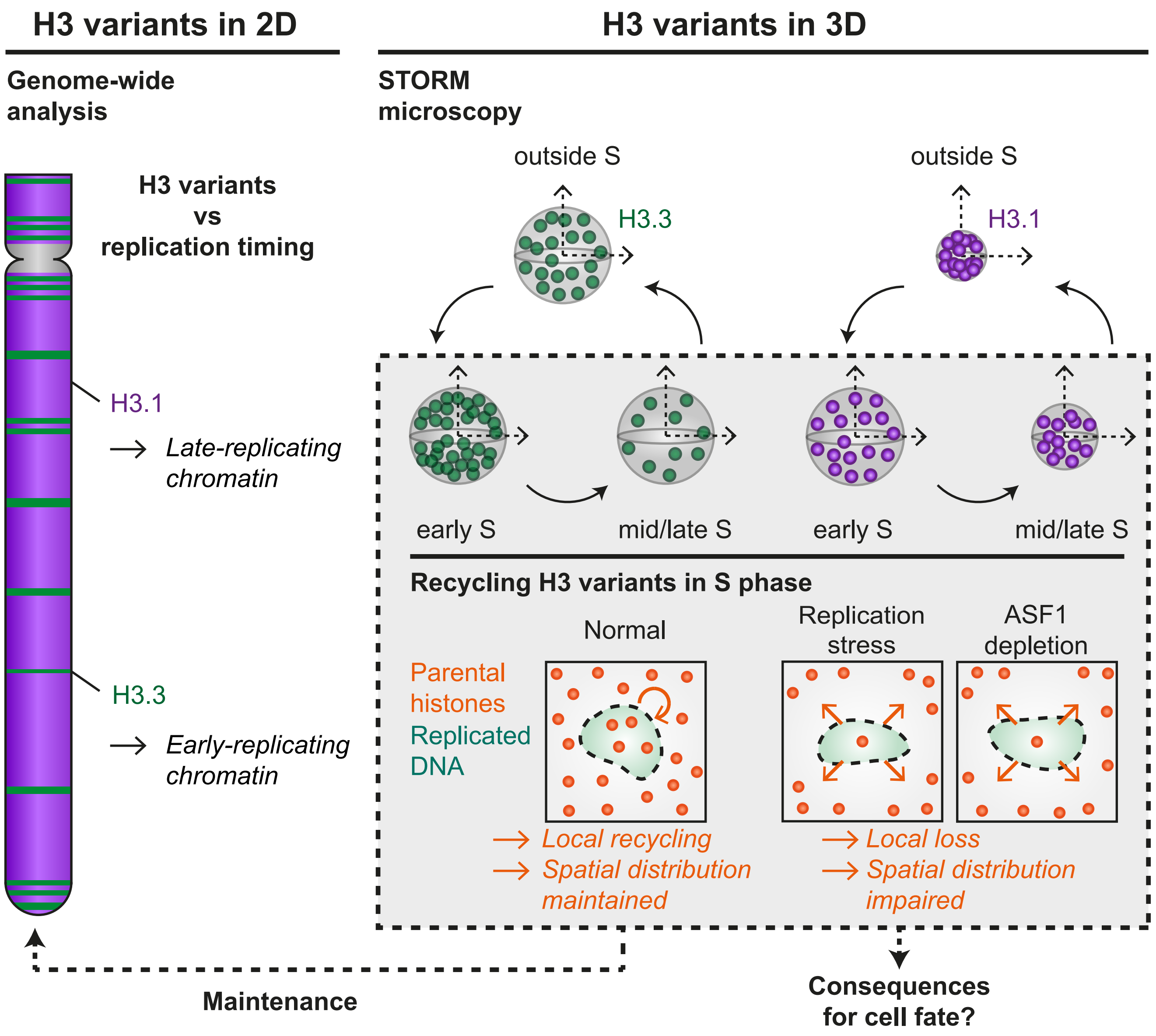

Supplementary Figure 18

Graphical abstract

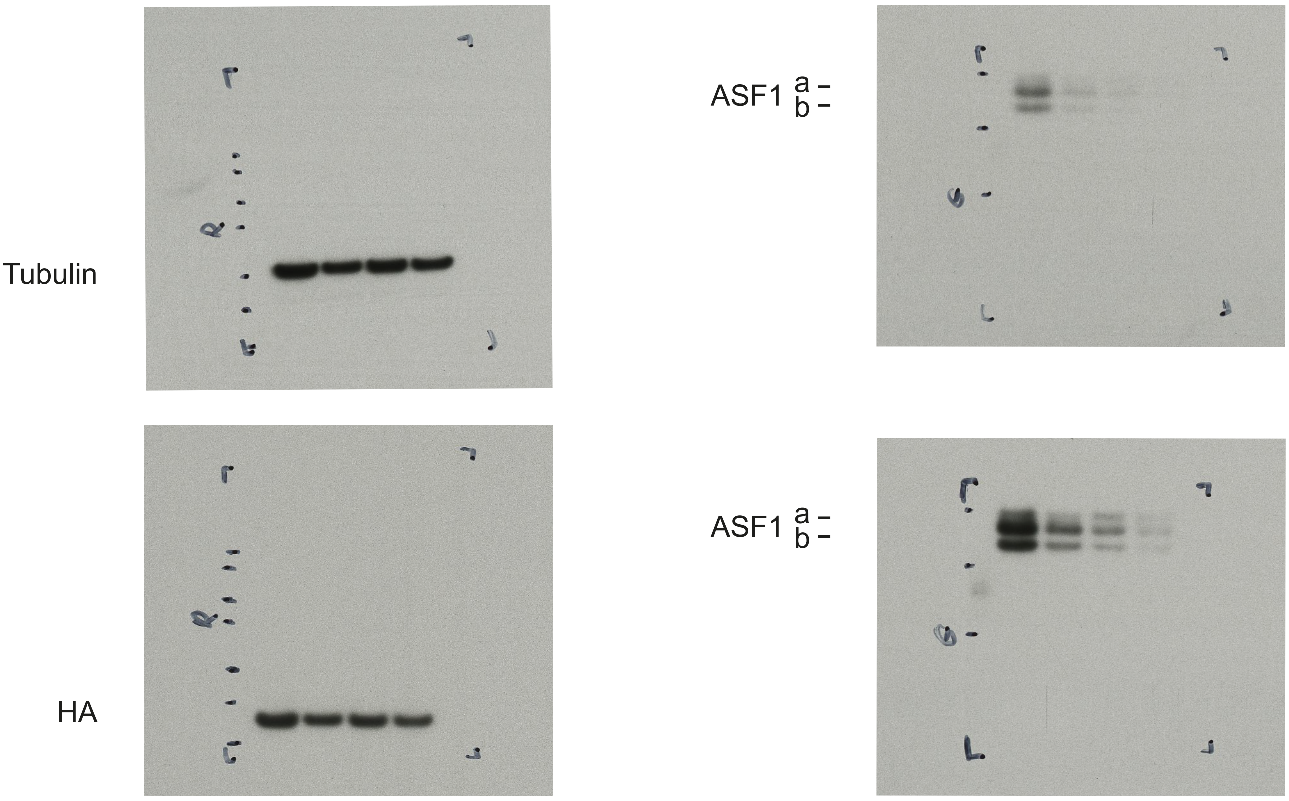

Supplementary Figure 19

Full images for Western Blot analysis of ASF1a and ASF1b knockdown efficiency using siRNA against ASF1 (see Supplementary Figure 9). The SNAP H3.1 also contains a HA-tag, which was probed for in this Western Blot.
